# Lingual Frenectomy in 3–9-Year-Old Children Using Diode Laser: A Case Series

**DOI:** 10.7759/cureus.97893

**Published:** 2025-11-26

**Authors:** Supriya H Solanke, Ashwin Jawdekar, Laresh N Mistry, Swati Kale, Tanvi Saraf, Farheen Tafti

**Affiliations:** 1 Department of Pediatric and Preventive Dentistry, Bharati Vidyapeeth (Deemed to be University) Dental College and Hospital, Navi Mumbai, IND

**Keywords:** ankyloglossia in children, frenotomy, laser frenectomy, tongue tie, tongue tie management

## Abstract

Ankyloglossia, or tongue-tie, not only causes speech impairment but also causes problems like difficulty in breastfeeding in infants, restricts tongue movement, which affects its mobility to clean teeth, restricts mandibular growth, and results in a reverse swallow pattern. Conventional surgical techniques for frenectomy can be difficult in children. However, with diode laser, there is controlled bleeding and surgery is painless. The purpose of this article is to present five cases of lingual frenectomy using the diode laser in young children with moderate-severe ankyloglossia. Five children aged 3-9 years with a short lingual frenum, restricted tongue movement, impaired speech, and reverse swallowing pattern were treated using a laser frenectomy procedure. Complete blood count and bleeding time, clotting time tests were carried out in all the patients before treatment. Tongue mobility exercises were taught to patients, and follow-up was taken till recovery and for one year. A satisfactory outcome of tongue mobilization was evident in four cases, but in one case, reattachment/adhesion was noticed six months after the frenulum was treated with laser tongue-tie release.

## Introduction

Ankyloglossia, commonly known as tongue-tie, refers to a restriction in tongue mobility due to a shortened or tight lingual frenulum [1]. This condition can interfere with various oral functions in children, including speech articulation, social communication, tongue movement, oral hygiene, and swallowing [2]. The prevalence of ankyloglossia in infants and using different assessment tools is 5% reported by Cruz et al [3]. In children aged < 1 year, according to Hill et al. (2020), the incidence is 8%, and it is more frequently observed in males, with a male-to-female ratio of approximately 2-3:1 [4,5].

Classification of tongue-tie can be challenging and is often based on both anatomical position and degree of mobility limitation. It is typically categorized into anterior and posterior types. Anterior tongue-tie usually involves the frenulum attaching near the tip of the tongue, while posterior tongue-tie may involve deeper submucosal bands that limit movement, although consensus on its exact definition remains inconsistent among clinicians. Classification of ankyloglossia according to Kotlow (based on the “free tongue” length) is given as: Normal, clinically acceptable range of “free tongue” >16 mm. Class 1: mild ankyloglossia 12-16 mm, Class II: moderate ankyloglossia 8-11 mm, Class III: severe ankyloglossia 3-7 mm, Class IV: complete ankyloglossia<3 mm [6,7]. Ankyloglossia may also be associated with various genetic and congenital syndromes, including Ehlers-Danlos Syndrome, Beckwith-Wiedemann Syndrome, Simosa Syndrome, X-linked cleft palate, and orofaciodigital syndrome [8]. EDS, in particular, offers important insights as it includes multiple hereditary connective tissue disorders that may impact oral structures, including the lingual frenulum [9].

The primary treatment for ankyloglossia is frenectomy, a surgical intervention aimed at releasing the restricted frenulum to restore normal tongue mobility [2]. This can be performed using either conventional surgical methods involving scissors or a scalpel, followed by suturing, or with laser technology. Laser-assisted frenectomy has gained popularity due to its precision, minimal bleeding, reduced postoperative discomfort, and faster healing. Shorter operative working time, tissue cauterization and sterilization, hemostasis is achieved easily, less local anesthesia requirement, and fewer postoperative complications (pain, swelling, and infection). Laser also enhances access and visualization due to the lack of interposed instruments and bleeding in the operative field [6].

Additionally, the need for suture is eliminated, and a uniform depth in the surgical site is maintained, reducing unnecessary damage to the tongue muscle. For all these features, laser is well tolerated by children [10]. Diode or high-power lasers are especially advantageous due to their tissue-specific wavelength and minimal thermal damage, providing improved surgical outcomes and enhanced patient comfort during recovery [11]. The objective of this case series is to report five cases of lingual frenectomy with diode low-power laser.

## Case presentation

Case 1

An eight-year-old male patient presented to the Department of Pediatric Dentistry of Bharati Vidyapeeth Dental College and Hospital, Navi Mumbai, with a chief complaint of tooth pain ( reversible pulpitis with respect to 75). On examination, the patient was able to protrude his tongue beyond the lips and approximately one-third of the distance down the chin. While tongue extension appeared relatively normal, he was unable to achieve adequate elevation to contact the palate with his mouth fully open (Grade 2 Jose Duran tongue mobility classification).

**Figure 1:**
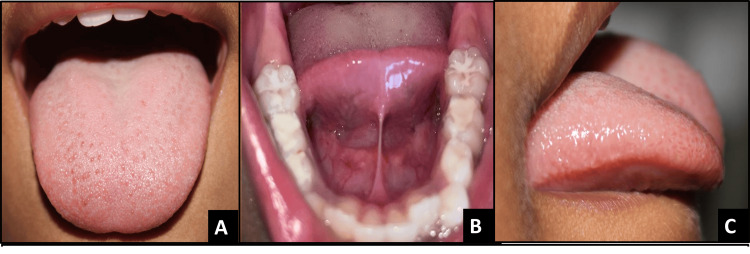
Pre-operative images of case 1 A - Dorsal view, B - Appearance when lifted, C - Lateral view

He had a short frenum and a slight cleft on the protrusion of the tongue. The patient was diagnosed with moderate ankyloglossia, with a free tongue length of 10 mm, as shown in Figures 1A-1C. Dental treatment for 75 was provided first, and then a frenectomy was performed. The treatment was uneventful. The patient remained comfortable and cooperative throughout the procedure. At the 1-week follow-up, the patient reported no pain and demonstrated increased lingual elevation, at one-month and one-year follow-up, complete release of tongue-tie can be seen with satisfactory wound healing with no signs of infection or complications. Post-op free tongue length measured 17 mm.

Case 2

A nine-year-old female patient presented to the Department of Pediatric Dentistry of Bharati Vidyapeeth Dental College and Hospital, Navi Mumbai, with a chief complaint of tooth pain. Clinical examination revealed a heart-shaped appearance of the tongue upon protrusion, restricted tongue mobility, and a reverse swallowing pattern, and the patient misarticulated the speech sounds /s/, /r/, and /ch/.

**Figure 2:**
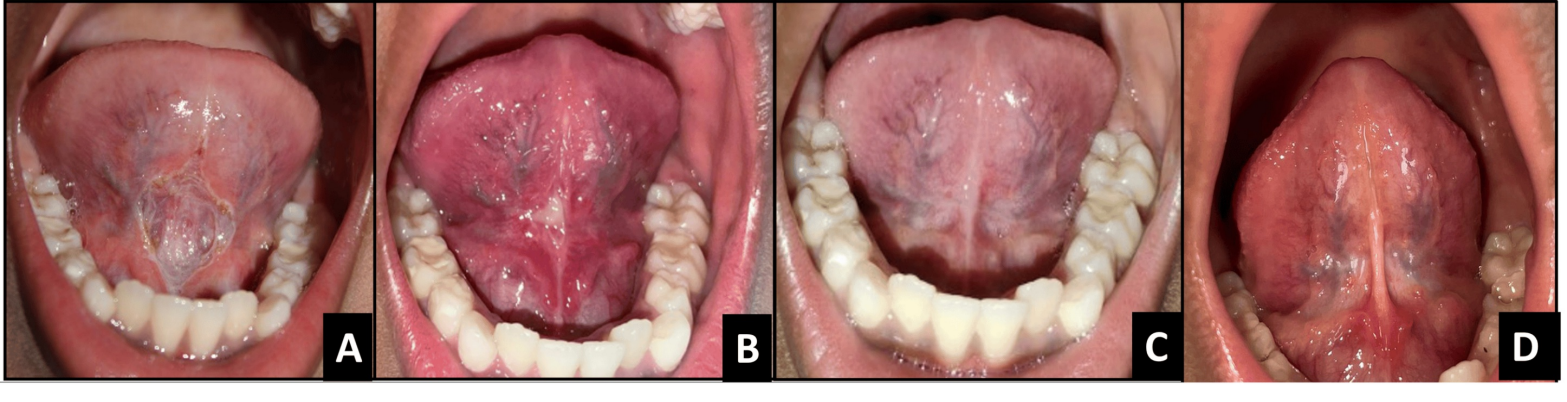
Postoperative images of case 1 A - immediately after surgery, B - 8-day follow-up, C - one-month follow-up, D - one-year follow-up

The patient was diagnosed with moderate ankyloglossia, with a free tongue length of 9 mm. Frenum was short, thick, and fibrous as shown in Figures 2A-2C. The treatment was uneventful (postoperative period without any complications, such as excessive bleeding, infection, delayed healing, pain beyond expected levels, or reattachment of the frenum. It also indicates that the child tolerated the procedure well, healing progressed normally, and routine postoperative care was sufficient. Initially, the patient exhibited uncooperative behavior; however, using the Tell-Show-Do technique, effective cooperation was successfully achieved. In this case, a 1-week follow-up was not conducted because the patient did not return for the scheduled visit despite reminders. At the 1-month follow-up, the patient reported no pain, wound healing with no signs of infection or complications, post-op free tongue length measured 16 mm, and demonstrated increased lingual elevation; therefore, the patient is referred to a speech therapist, and at 1-year follow-up, a satisfactory outcome is seen.

Case 3

**Figure 3:**
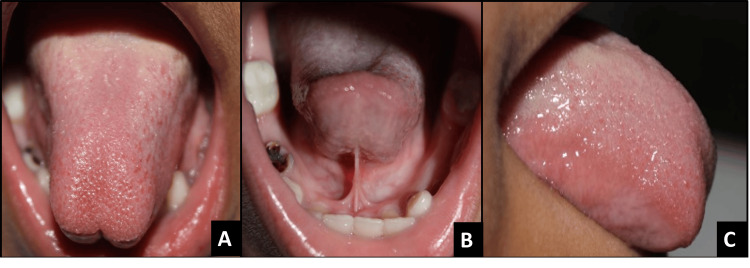
Pre-operative images of case 2 A - Dorsal view, B - Appearance when lifted, C - Lateral view

A six-year-old male patient presented to the Department of Pediatric Dentistry of Bharati Vidyapeeth Dental College and Hospital, Navi Mumbai. Clinical examination revealed a heart-shaped appearance of the tongue upon protrusion, restricted tongue mobility, and slurred speech (stuttering and mumbling). The patient was diagnosed with severe and posterior ankyloglossia, with a free tongue length of 6 mm, as shown in Figures 3A-3C. The patient remained comfortable and cooperative throughout the procedure. In this patient, an extensive release of the lingual frenulum was performed due to the severity of the restriction. The patient reported pain during the procedure, following which an additional amount of local anesthetic was administered to ensure comfort.

The patient was non-compliant with the prescribed tongue exercises and demonstrated limited tongue movement during follow-up. The patient is referred to a speech therapist for speech improvement. A 1-week follow-up showed suboptimal healing and limited functional improvement. Although the surgical site had begun to epithelialize normally, the child demonstrated restricted tongue elevation, mild discomfort during movement, and poor compliance with the prescribed myofunctional exercises. Early signs of partial reattachment were also noted, likely due to inadequate postoperative stretching and limited wound mobilization. At the six-month follow-up, the patient reported a small swelling on the floor of the mouth adjacent to Wharton’s duct, attributed to partial ductal obstruction. The swelling subsequently ruptured spontaneously. Clinical examination also revealed reattachment and adhesion of the lingual frenum. The frenum appeared thicker, more fibrous, and shorter than before, indicating a recurrence; post-op free tongue length measured 8 mm. Based on these findings, the case was considered a treatment failure.

Case 4

**Figure 4:**
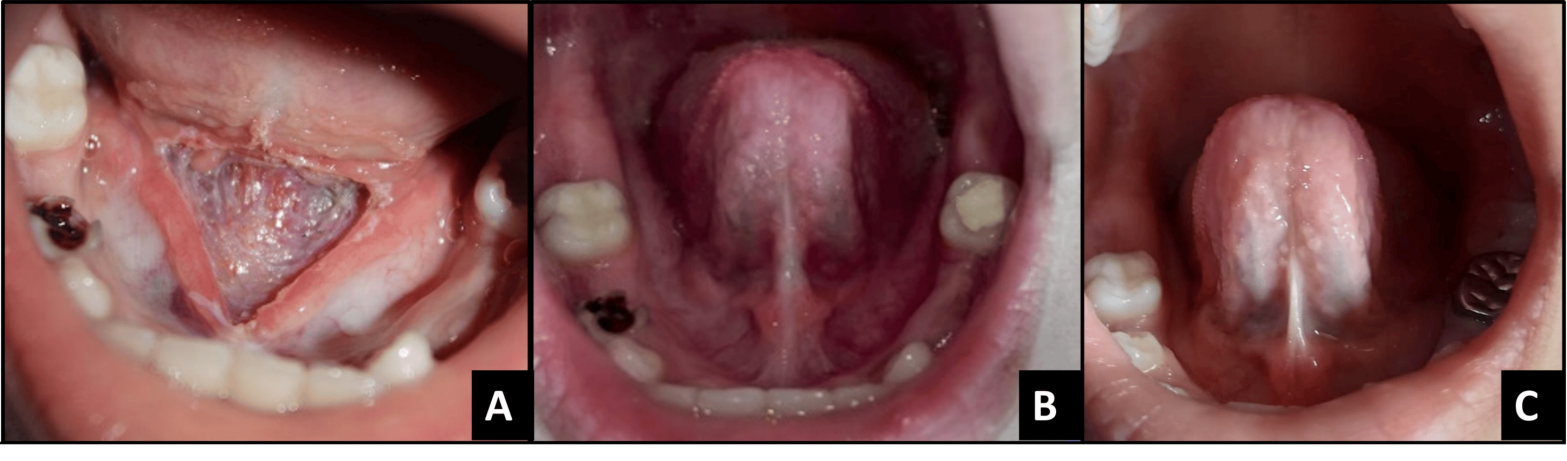
Postoperative images of case 2 A - immediately after surgery, B - one-month follow-up, C - one-year follow-up

A 4-year-old boy presented to the Department of Pediatric Dentistry of Bharati Vidyapeeth Dental College and Hospital, Navi Mumbai. Clinical examination revealed severe early childhood caries, a heart-shaped appearance of the tongue upon protrusion, restricted tongue movements with severe ankyloglossia (free tongue length - 7 mm) as shown in Figures 4A-4C. patient misarticulated the speech sounds /s/, /r/, and /ch/. Treatment was uneventful; the patient was seen anxious before the procedure; however, using the Tell-Show-Do technique, effective cooperation was successfully achieved.

At the one-month follow-up, the patient reported no pain and demonstrated improved lingual elevation, post-op free tongue length measured 13 mm. Wound healing was satisfactory, with no signs of infection or complications observed. However, the patient is referred to a speech therapist, and long-term follow-up is recommended to monitor the stability of the outcome.

Case 5

**Figure 5:**
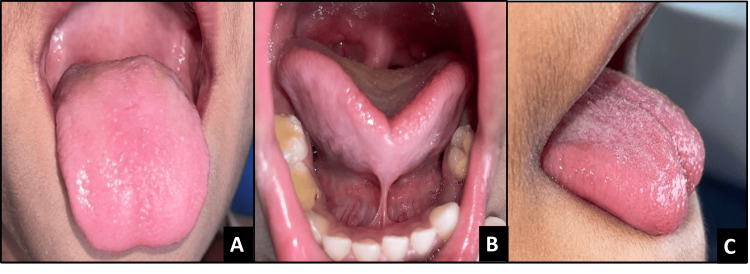
Pre-operative images of case 3 A - Dorsal view, B - Appearance when lifted, C - Lateral view

A 3-year-old boy presented to the Department of Pediatric Dentistry of Bharati Vidyapeeth Dental College and Hospital, Navi Mumbai. Examination revealed he had a cleft at the tip of the tongue on protrusion, restricted tongue movements, and a thick and fibrous frenulum with moderate ankyloglossia (free tongue length - 8 mm) as shown in Figures 5A-5C. The patient had decayed lower anterior teeth (severe early childhood caries).

Treatment was uneventful; the patient was seen anxious before the procedure; however, using the Tell-Show-Do technique, effective cooperation was successfully achieved. At the two-week follow-up, the patient reported no pain and demonstrated increased lingual elevation. At one-month follow-up, a satisfactory outcome and wound healing with no signs of infection or complications were seen. Post-op, the free tongue length was measured at 14 mm. Long-term follow-up is needed.

Technique

**Figure 6:**
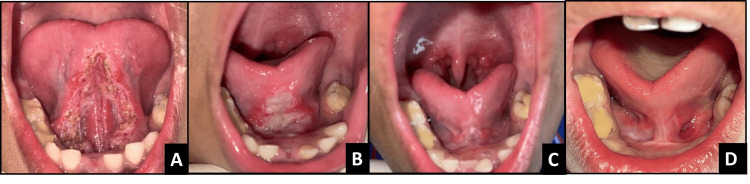
Postoperative images of case 3 A - immediately after surgery, B - 8-day follow-up, C - one-month follow-up, D - six-month follow-up

Prior to the procedure, patients were advised to undergo a complete blood count along with bleeding time and clotting time assessments. Written informed consent was obtained from the parents. Topical anesthetic gel (Septodont) was applied, followed by local infiltration using 2% lidocaine with 1:100,000 epinephrine near the frenal attachments as shown in Figure 6. For safety, protective goggles were worn by the operator, patient, and assistant, and the laser tip was then activated (initiated) in order to cut the tissues.

**Figure 7:**
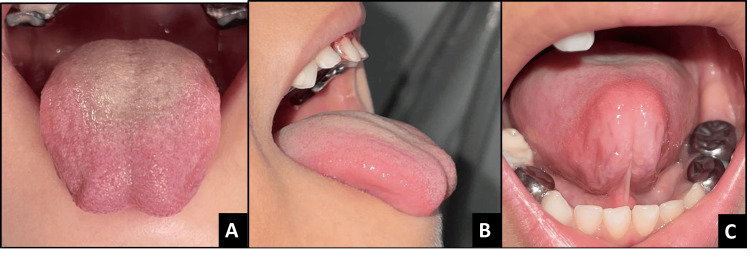
Pre-operative images of case 4 A - Dorsal view, B - Lateral view, and C - Appearance when lifted

**Figure 8:**
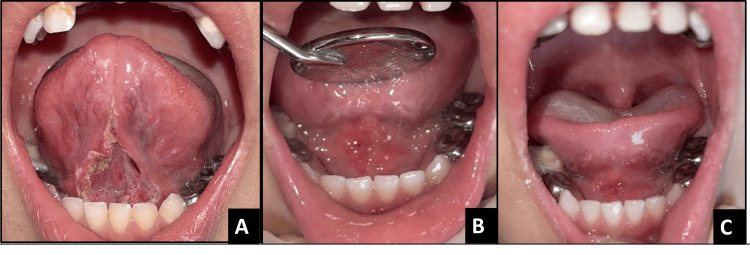
Postoperative images of case 4 A - immediately after surgery, B and C - one-month follow-up

**Figure 9:**
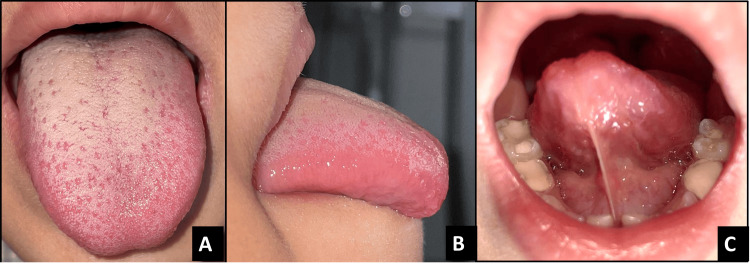
Pre-operative images of case 5 A - Dorsal view, B - Lateral view, C - Appearance when lifted

**Figure 10:**
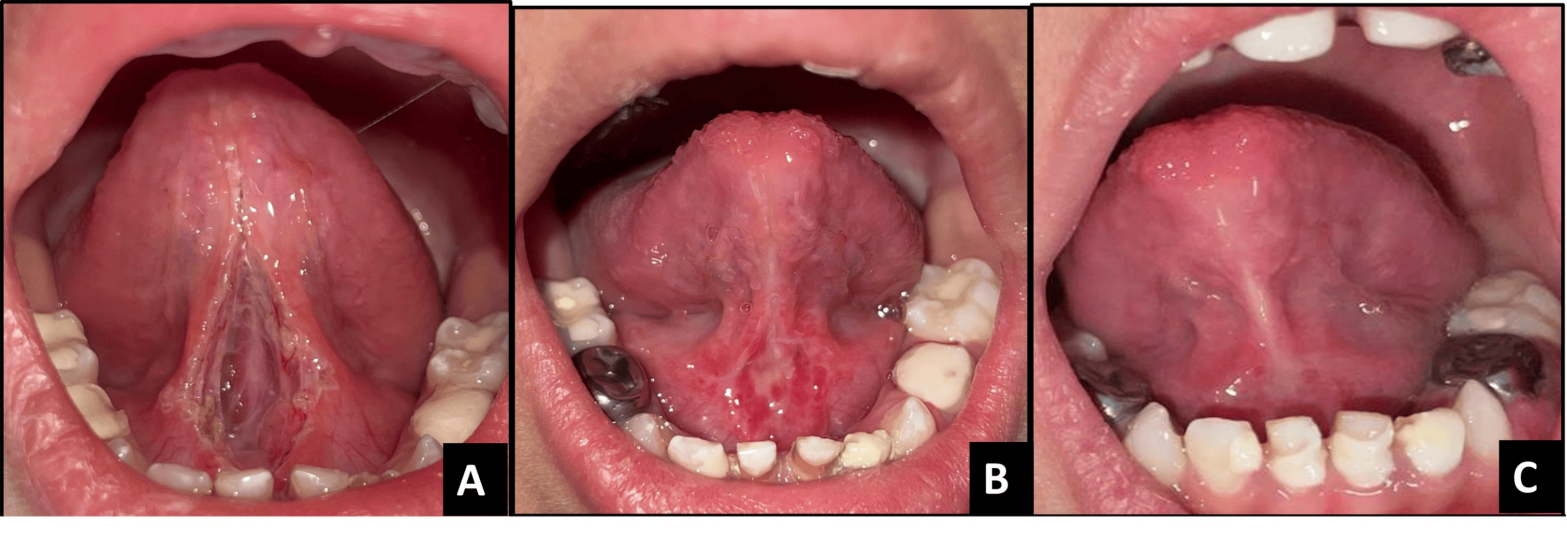
Postoperative images of case 5 A - immediately after surgery, B - 8-day follow-up, C - one-month follow-up

**Figure 11:**
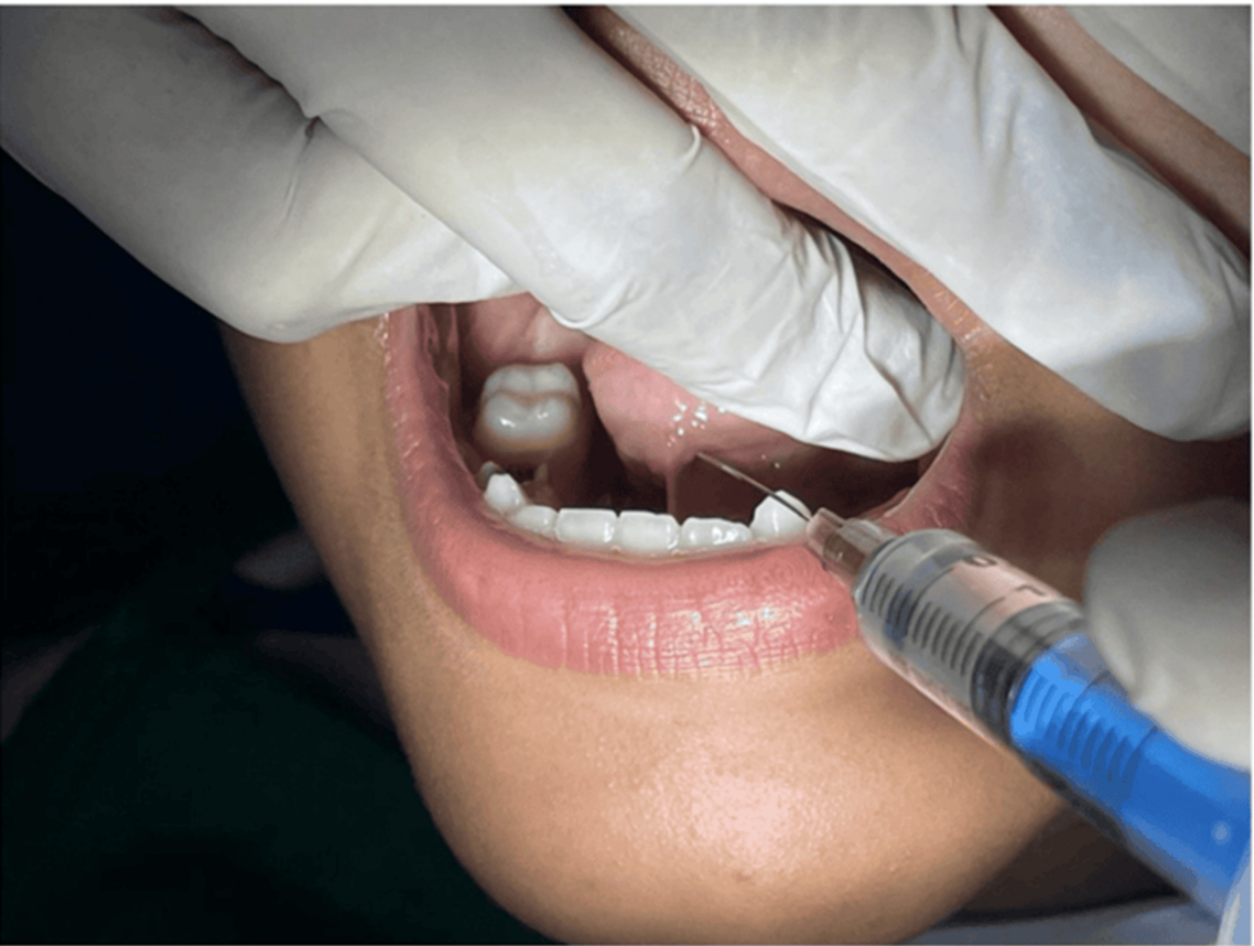
Administration of local infiltration anesthesia

**Figure 12:**
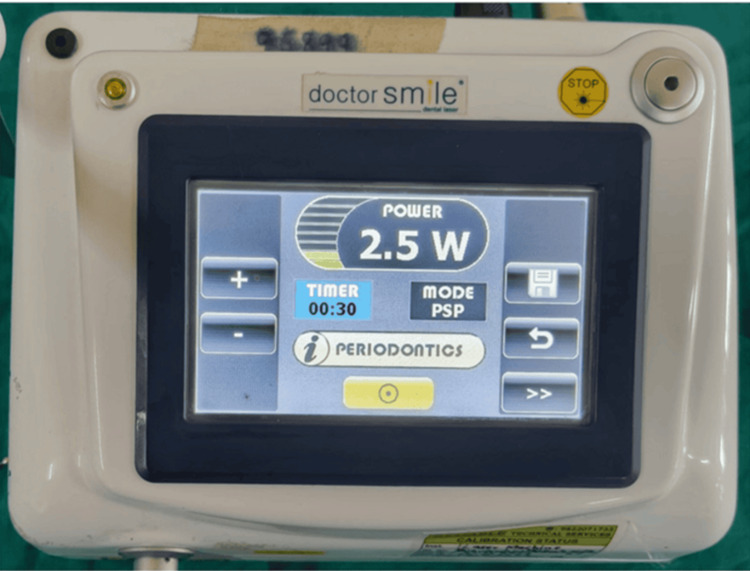
Diode laser set at 2.5 W power

**Figure 13:**
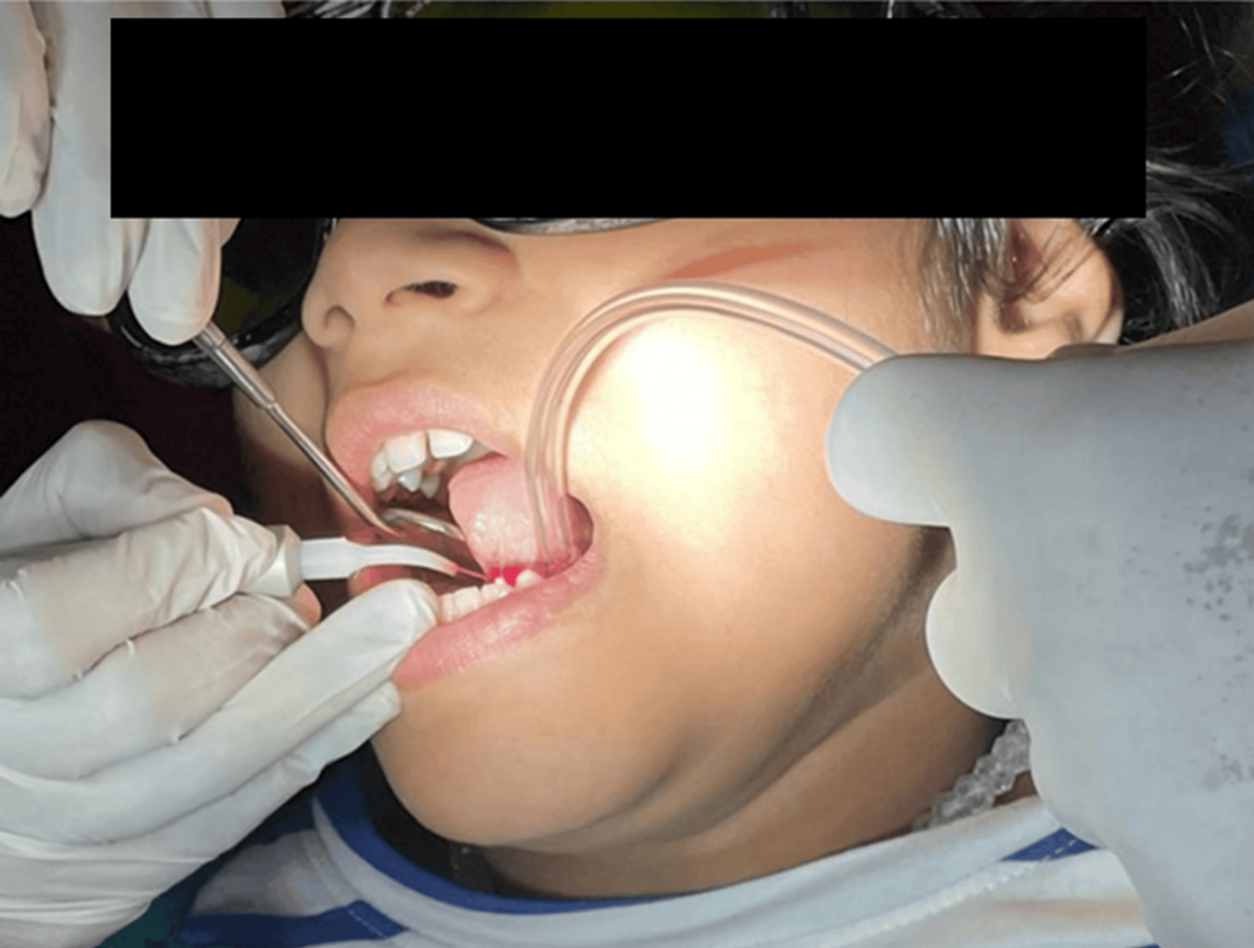
Vertical strokes to open up the frenum

Frenectomy was performed using a diode laser with a wavelength of 810 nm, set to a power output of 2.5-3.0 W in continuous mode, as shown in Figures 7-8. The tongue was elevated using a gauze piece and fingers, and vertical strokes were applied from the tip to the floor of the mouth to release the frenum, followed by horizontal strokes to complete the excision. No bleeding was observed due to the coagulative effect of the laser, and sutures were not required. Postoperatively, patients were instructed in tongue exercises, namely side-to-side movement, tongue lifting, and stretching, and advised to perform them 5-6 times daily for five minutes each session. Antibiotics and analgesics were prescribed as part of the postoperative care. Postoperative images of all the patients are shown in Figures 9-13, respectively.

## Discussion

Over the past decade, there has been a growing awareness and increased surgical management of ankyloglossia in pediatric populations [10]. However, the relationship between tongue-tie and speech articulation difficulties remains unclear, as much of the available literature is based on small sample sizes, lacks control groups, or fails to employ standardized objective measures [12,13].

Consequently, despite clinical observations, definitive conclusions regarding the impact of tongue-tie on speech remain inconclusive. The etiology of ankyloglossia is not fully understood, though it is believed to result from aberrant development of the mucosa over the anterior two-thirds of the mobile tongue during embryogenesis [5]. In most cases, it appears as an isolated anatomical variation rather than a component of a syndromic condition. Nonetheless, ankyloglossia has been associated with several genetic syndromes, including Ehlers-Danlos Syndrome and Beckwith-Wiedemann Syndrome [10].

A comprehensive assessment should include both morphological and functional evaluations of the tongue. Surgical intervention is generally recommended when the functional limitations, such as impaired speech or oral hygiene, can be clearly linked to the lingual frenulum and are likely to be improved through frenectomy [7]. Restricted tongue mobility in ankyloglossia can interfere with the production of certain linguodental and alveolar consonants, such as /t/, /d/, /n/, and /l/, potentially leading to articulation disorders, including lisping [14]. Additionally, prolonged tongue restriction may contribute to malocclusion, gingival recession, and abnormal mandibular growth, especially when not addressed early [15].

Although the lingual frenulum tends to elongate naturally as the mandible develops and teeth erupt between the ages of 2 and 5 years, some children continue to experience functional impairments [2]. Surgical consultation is often sought for concerns such as feeding difficulties, airway obstruction, speech delay, or periodontal issues. Tongue-tie disrupts proper tongue rest posture, and this contributes to orofacial myofunctional disorders, further leading to a narrow palate, constricted arches, crowding, and spacing. Ankyloglossia may affect occlusion and craniofacial growth, as low tongue posture is linked to Class III malocclusion. Correcting tongue position can improve hyoid alignment and reduce mandibular tension, while a low tongue rest may cause clockwise jaw rotation, contributing to an anterior open bite [16, 17].

Frenectomy can be performed via traditional scalpel techniques or with LASER-assisted surgery. Laser procedures are increasingly favored in pediatric settings due to their minimal invasiveness, reduced bleeding, lower pain perception, and faster healing times [18]. Specifically, diode LASERs offer excellent precision, coagulative ability, and bactericidal effects, making them ideal for treating soft tissue anomalies like tongue-tie [6]. In the present case series, five children aged 3-9 years with varying severity of ankyloglossia underwent lingual frenectomy using a diode LASER. The outcomes observed were uniformly positive, with minimal intraoperative bleeding, no need for suturing, rapid healing, and improved tongue mobility postoperatively. Traditional frenectomy with scalpel or scissors often results in profuse bleeding, a need for sutures, increased operative time, and considerable discomfort in pediatric patients [5].

In contrast, LASER-assisted frenectomy offers several advantages, including: Precise soft tissue ablation, minimal bleeding due to coagulation effect, reduced postoperative edema and pain, no sutures required, faster healing, and better patient cooperation [6,7]. The diode laser used in this study (2.5-3.0 W, continuous mode) enabled safe and efficient soft tissue incision with minimal collateral damage. This aligns with findings by Kotlow, who emphasized diode lasers’ effectiveness for soft tissue procedures in children due to their high absorption in melanin and hemoglobin [6]. Although classification systems for tongue-tie (like Coryllos or Kotlow) exist, they often lack consensus, particularly in diagnosing posterior ankyloglossia [14]. Nevertheless, the functional restriction seen in these cases, such as limited protrusion, reverse swallowing pattern, and slurred speech, clearly indicated the need for surgical intervention.

Postoperative outcomes

All five patients exhibited satisfactory wound healing with no signs of infection or complications. Follow-up was conducted for a minimum of one month to assess wound healing, though no standardized index was used. Healing was evaluated based on clinical observation, including visual inspection for absence of infection, swelling, discharge, or hemorrhage. Palpation was performed to check for tenderness or signs of delayed healing. Patient-reported outcomes such as reduced pain, improved comfort, and no difficulty in eating or speaking were also considered. By one month, return to normal function and scar remodeling were expected. Tongue mobility exercises initiated immediately post-procedure helped in preventing reattachment and promoting function, corroborating recommendations from past studies [14].

It is noteworthy that early surgical correction during the developmental years, especially before 9 years, can enhance oral motor function and potentially minimize the need for future speech therapy or orthodontic correction [2]. Despite its benefits, one limitation of frenectomy is the potential for reattachment of the frenum. This often results from inadequate postoperative care or lack of compliance with prescribed tongue mobility exercises.

In our series, the third case of a 6-year-old boy demonstrated less favorable outcomes and formation of thick fibrous frenum reattachment due to poor adherence to postoperative physiotherapy protocols, underscoring the importance of patient and caregiver education in achieving optimal results. To address these issues, detailed parent and patient counseling was provided, emphasizing the importance of rehabilitation, wound care, and consistent follow-up. The patient was instructed to perform specific myofunctional exercises, including tongue elevation, protrusion, and swallow retraining. Additionally, we recognize that suturing after the procedure could have contributed to improved wound stability, reduced reattachment risk, and facilitated better functional recovery in this particular case. Closer follow-up visits were arranged to monitor healing, reinforce exercise performance, and ensure compliance.

## Conclusions

This case series highlights the clinical effectiveness and advantages of diode laser-assisted lingual frenectomy in managing moderate to severe ankyloglossia in children aged 3-9 years. The use of a diode laser allowed for precise soft tissue excision with minimal intraoperative bleeding, no need for suturing, reduced postoperative discomfort, and faster healing. Four out of five cases demonstrated successful improvement in tongue mobility and overall oral function, with no major complications reported. However, the study also emphasizes that successful outcomes depend not only on the surgical technique but also on consistent postoperative care and adherence to tongue mobility exercises. The recurrence of tongue-tie observed in one patient due to poor compliance highlights the critical role of patient and caregiver education in maintaining treatment results. Diode laser frenectomy, combined with comprehensive follow-up and interdisciplinary support, such as speech therapy when indicated, offers a safe and effective treatment modality for pediatric ankyloglossia. Long-term monitoring is essential to ensure stability and identify any signs of reattachment or functional regression.
